# Progressive Rise in Red Blood Cell Distribution Width Predicts Mortality and Cardiovascular Events in End-Stage Renal Disease Patients

**DOI:** 10.1371/journal.pone.0126272

**Published:** 2015-05-11

**Authors:** Hye Eun Yoon, Sung Jun Kim, Hyeon Seok Hwang, Sungjin Chung, Chul Woo Yang, Seok Joon Shin

**Affiliations:** 1 Division of Nephrology, Department of Internal Medicine, College of Medicine, The Catholic University of Korea, Seoul, Korea; 2 Department of Internal Medicine, Incheon St. Mary’s Hospital, Incheon, Korea; Chi-Mei Medical Center, TAIWAN

## Abstract

Red blood cell distribution width (RDW) is a robust marker of adverse clinical outcomes in various populations. However, the clinical significance of a progressive rise in RDW is undetermined in end-stage renal disease (ESRD) patients. The purpose of this study was to determine the prognostic importance of a change in RDW in ESRD patients. Three hundred twenty-six incident dialysis patients were retrospectively analyzed. Temporal changes in RDW during 12 months after dialysis initiation were assessed by calculating the coefficients by linear regression. Patients were divided into two groups: an RDW-decreased group who had negative coefficient values (n = 177) and an RDW-increased group who had positive values (n = 149). The associations between rising RDW and mortality and cardiovascular (CV) events were investigated. During a median follow-up of 2.7 years (range, 1.0–7.7 years), 75 deaths (24.0%) and 60 non-fatal CV events (18.4%) occurred. The event-free survival rate for the composite of end-points was lower in the RDW-increased group (*P* = 0.004). After categorizing patients according to baseline RDW, the event-free survival rate was lowest in patients with a baseline RDW >14.9% and increased RDW, and highest in patients with a baseline RDW ≤14.9% and decreased RDW (*P* = 0.02). In multivariate analysis, rising RDW was independently associated with the composite of end-points (hazard ratio = 1.75, *P* = 0.007), whereas the baseline RDW was not. This study shows that a progressive rise in RDW independently predicted mortality and CV events in ESRD patients. Rising RDW could be an additive predictor for adverse CV outcomes ESRD patients.

## Introduction

Red blood cell distribution width (RDW) is a quantitative measure of the size variability of circulating erythrocytes and is reported as part of the standard complete blood cell count in clinical practice. Classically, RDW is elevated in conditions of ineffective erythropoiesis or increased erythrocyte destruction [1]. In recent years, research has demonstrated the significance of RDW as a prognostic marker for cardiovascular (CV) morbidity and mortality in various diseases, including heart failure [2, 3], coronary artery disease [4], stroke [5], and diabetes [6], and in community-based cohorts [7, 8]. The risk of mortality is high in patients with end-stage renal disease (ESRD) [9], and CV diseases are the leading cause of mortality in these patients [10]. However, there are few data on the association between RDW and adverse outcomes in ESRD patients. Because acute changes in RDW may result from blood loss or haemolysis, the RDW value may fluctuate during the early period after dialysis. We hypothesized that a progressive rise in RDW rather than the baseline RDW value would be associated with adverse outcomes in ESRD patients. This study evaluated the association between rising RDW and death and CV events in ESRD patients starting dialysis at a single centre in Korea.

## Materials and Methods

### Study population

Three hundred thirty-seven ESRD patients who initially started maintenance haemodialysis (HD) or peritoneal dialysis (PD) between January 2006 and June 2012 were enrolled. The inclusion criteria were incident dialysis patients ≥18 years of age who received dialysis for >3 months. Patients referred from other centres and those lacking consecutive RDW measurements within 3 months after initiating dialysis were excluded. Eleven patients who died or suffered from CV event within one year after dialysis initiation were excluded. A total of 326 patients were included in this study. In line with the principles of the Declaration of Helsinki, the study protocol was approved by the institutional review board of the Catholic University of Korea (OC14RISE0075). A written consent was not obtained but the patient records/information was anonymized and de-identified prior to analysis.

### Data collection and definition

The medical records of the patients were reviewed retrospectively by a third party person blinded to this study. The baseline demographics and baseline RDW values were collected at the start of dialysis. The reference range for RDW in our laboratory is 11.6–14.9%. The time-averaged RDW values were calculated as the average of the individual RDW values obtained at regular intervals within the first year of dialysis initiation. The change in RDW was calculated from the regression coefficient between RDW and time, the first year of dialysis initiation. The RDW-slope was defined as the regression coefficient value. The RDW-decreased group was defined as patients with negative regression coefficient values, and the RDW-increased group as those with positive regression coefficient values.

Other laboratory data were collected as time-averaged values during the first year of dialysis initiation. Vitamin B12 levels were missing in 42.9% (n = 140) of patients, and folate levels were missing in 44.5% (n = 145). Echocardiography was performed within 3 months of dialysis initiation and on a non-dialysis day in HD patients. Echocardiographic data were missing in 27.9% (n = 91) of patients.

Haemoglobin variability was assessed by calculating the standard deviation (SD) of haemoglobin, the coefficient of variation (CV) of haemoglobin, slope of linear regression of haemoglobin values (hemoglobin-slope), and residual SD of haemoglobin [11, 12]. Residual SD of haemoglobin is the SD of the differences between observed haemoglobin values and the regression line.

Body mass index (BMI) was calculated as the ratio of weight in kilograms divided by the square of height in meters. The estimated glomerular filtration rate was calculated using the abbreviated Modification of Diet in Renal Disease formula [13]. The erythropoietin resistance index was calculated as the weekly weight-adjusted dose of erythropoietin (U/kg/week) divided by haemoglobin concentration (g/dL) [14].

### End-points

The end-points for the study were all-cause mortality and non-fatal CV events. CV events were defined as coronary artery disease (coronary artery bypass surgery, percutaneous intervention, or myocardial infarction), heart failure, ventricular arrhythmia, cerebrovascular accident (cerebral infarction, transient ischemic attack, or cerebral haemorrhage), or peripheral arterial disease (peripheral vascular revascularization or amputation).

### Statistical analysis

Data are expressed as the mean ± SD. Differences between the two groups were determined using Student’s *t* test or the Mann–Whitney *U* test, as appropriate. Categorical variables were compared using the chi-square test or Fisher’s exact test. Pearson’s correlation analysis or Spearman correlation analyses were used to determine the correlation between time-averaged RDW and time-averaged laboratory and echocardiographic measurements. Kaplan–Meier curves and log-rank tests were used to describe and compare the event-free survival rates for all-cause mortality and non-fatal CV events. A multivariate Cox regression analysis identified the significant prognostic factors affecting all-cause mortality and non-fatal CV events. Forward stepwise selection was used in Cox regression with the probability value for independent covariates to enter or stay in the model set at 0.05. The estimated standard error of the coefficient (β1) was used to establish the confidence interval of the hazard ratio (HR). A *P* value of <0.05 was considered significant. The statistical analyses were performed using SPSS software version 20.0 (SPSS, IBM Corp., Armonk, NY).

## Results

### Clinical characteristics and laboratory and echocardiographic measurements

Patients were divided into two groups according to the change in RDW values: an RDW-decreased group (n = 177) and an RDW-increased group (n = 149). Table 1 shows the baseline clinical characteristics of the study population. The RDW-increased group was older and the percentage of patients who received iron replacement was lower than did the RDW-decreased group. Other demographic parameters and use of medications did not differ between the two groups.

**Table 1:** Clinical characteristics of the study population.

	RDW- decreased (n = 177)	RDW-increased (n = 149)	*P*
Age (years)	55.1 ± 12.8	58.4 ± 12.5	0.02
Male (%)	93 (52.5)	83 (55.7)	0.57
Diabetes (%)	102 (57.6)	96 (64.4)	0.21
Cause of ESRD (%)			0.84
Diabetes	103 (58.2)	95 (63.8)	
Hypertension	45 (25.4)	36 (24.2)	
Chronic glomerulonephritis	20 (11.3)	7 (4.7)	
Others	9 (5.1)	11 (7.4)	
Previous CV disease (%)	33 (18.6)	38 (25.5)	0.14
History of smoking (%)	57 (32.2)	44 (29.5)	0.60
BMI (kg/m^2^)	23.7 ± 4.0	23.6 ± 4.2	0.97
Hemodialysis (%)	96 (54.2)	86 (57.7)	0.53
eGFR (mL/min/1.73m^2^)	8.9 ± 6.2	9.5 ± 4.4	0.34
Follow-up years	3.3 ± 1.7	3.0 ± 1.7	0.08
Antihypertensive drugs (%)			
RAS blockers	135 (76.3)	114 (76.5)	0.96
Beta-blockers	100 (56.5)	85 (57.0)	0.92
Calcium channel blockers	109 (61.6)	98 (65.4)	0.43
Phosphate binders (%)			
Calcium-based	87 (49.2)	66 (44.3)	0.38
Non calcium-based	19 (10.7)	14 (9.4)	0.69
Erythropoietin stimulating agent (%)	167 (94.4)	136 (91.3)	0.28
Erythropoietin resistance index (U/kg/week/g/dL)	12.5 ± 16.2	16.8 ± 34.4	0.17
Iron replacement (%)	151 (85.3)	112 (75.2)	0.02
Statin (%)	70 (39.5)	50 (33.6)	0.26
Vitamin D analogue (%)	31 (17.5)	30 (20.1)	0.55

ESRD, end-stage renal disease; CV, cardiovascular; BMI, body mass index; eGFR, estimated glomerular filtration rate; RAS, renin-angiotensin system.

Table 2 shows the laboratory and echocardiographic measurements. The RDW-increased group had a lower baseline RDW value and higher follow-up RDW value at 1 year than did the RDW-decreased group. Fewer patients in the RDW-increased group had a baseline RDW >14.9% compared with the RDW-decreased group: 7.4% (n = 11) vs. 35.6% (n = 63) (*P* < 0.001). However, the time-averaged RDW values did not differ between the two groups. The RDW-increased group had lower SD and CV of haemoglobin and total iron-binding capacity than the RDW-decreased group. The vitamin B12 and folate levels did not differ between the two groups. The echocardiographic parameters did not differ between the two groups; these included left ventricular (LV) mass index, left atrial diameter, LV ejection fraction, and the ratio of early mitral inflow velocity to peak mitral annulus velocity.

**Table 2:** Laboratory and echocardiographic measurements.

	RDW-decreased (n = 177)	RDW-increased (n = 149)	*P*
Baseline RDW (%)	14.8 ± 1.9	13.6 ± 1.1	<0.001
Follow-up RDW at 1 year (%)	13.5 ± 1.2	15.0 ± 1.6	<0.001
RDW-slope	-3.5 ± 3.6	3.6 ± 3.5	<0.001
SD of haemoglobin	1.4 ± 0.7	1.2 ± 0.5	0.02
CV of haemoglobin	0.14 ± 0.08	0.13 ± 0.06	0.03
Haemoglobin-slope	0.14 ± 0.17	0.12 ± 0.15	0.20
Residual SD of haemoglobin	1.11 ± 0.65	0.96 ± 0.51	0.03
Time-averaged laboratory data			
Haemoglobin (g/dL)	9.8 ± 0.9	9.8 ± 0.9	0.66
White blood cell count (10^3^/mm^3^)	7.8 ± 2.7	7.6 ± 2.3	0.54
Platelet count (10^3^/mm^3^)	218.3 ± 68.6	219.4 ± 73.8	0.89
RDW (%)	14.2 ± 1.2	14.4 ± 1.1	0.15
Iron (μg/dL)	70.1 ± 37.2	66.2 ± 36.2	0.35
TIBC (μg/dL)	216.4 ± 44.0	205.0 ± 44.0	0.03
Transferrin saturation (%)	32.2 ± 16.3	33.7 ± 18.9	0.48
Ferritin (ng/mL)	286.3 ± 315.6	328.1 ± 495.9	0.37
Vitamin B12 (pg/mL)^a^	804.9 ± 332.5	1013.7 ± 1274.8	0.11
Folate (ng/mL)^b^	13.4 ± 12.9	17.3 ± 20.3	0.14
Albumin (g/dL)	3.7 ± 1.3	3.7 ± 1.4	0.80
Total cholesterol (mg/dL)	175.1 ± 54.0	181.7± 58.5	0.30
Triglyceride (mg/dL)	152.5 ± 89.8	175.4 ± 146.0	0.09
LDL-cholesterol (mg/dL)	108.0 ± 37.4	117.7 ± 113.6	0.37
Calcium (mg/dL)	8.0 ± 0.9	8.2 ± 0.8	0.09
Phosphorus (mg/dL)	5.4 ± 1.6	5.0 ± 1.3	0.07
Log CRP (mg/dL)	0.7 ± 0.6	0.7 ± 0.6	0.44
Intact PTH (pg/mL)	272.9 ± 217.6	244.7 ± 174.4	0.21
Echocardiographic data^c^			
LV mass index (g/m^2.7^)	56.2 ± 45.0	58.0 ± 38.5	0.74
LA diameter (mm^2^)	41.2 ± 5.9	41.9 ± 6.9	0.48
LV ejection fraction (%)	55.7 ± 12.5	56.4 ± 12.0	0.71
E/E’ ratio	14.5 ± 6.4	14.3 ± 5.5	0.84

RDW, red blood cell distribution width; TIBC, total iron binding capacity; LDL-cholesterol, low-density lipoprotein cholesterol; CRP, C-reactive protein; PTH, parathyroid hormone; LV, left ventricle; LA, left atrium, E/E’ ratio, early mitral inflow velocity to peak mitral annulus velocity ratio.

^a^n = 186;

^b^n = 181;

^c^n = 235.

### Correlations between time-averaged RDW values and other variables

Table 3 shows the correlation coefficients between time-averaged RDW values and time-averaged laboratory values and echocardiographic parameters. In the total population, there was a positive correlation between time-averaged RDW values and the time-averaged log of the C-reactive protein (CRP) concentration, and negative correlations between time-averaged RDW values and time-averaged haemoglobin and iron levels. In the RDW-increased group, time-averaged RDW values correlated positively with the time-averaged log CRP concentration and LV mass index, and negatively with time-averaged haemoglobin, total cholesterol, and serum iron levels, transferrin saturation, and LV ejection fraction. By contrast, in the RDW-decreased group, there were no significant correlations between time-averaged RDW values and other variables.

**Table 3:** Correlations between time-averaged RDW value and other parameters.

Variables	Total population (n = 326)		RDW-decreased (n = 177)		RDW-increased (n = 149)	
	*r*	*P*	*r*	*P*	*r*	*P*
Time-averaged values						
Log CRP	0.15	0.006	0.08	0.28	0.23	0.005
Haemoglobin	-0.16	0.004	-0.13	0.08	-0.19	0.02
Albumin	-0.06	0.27	-0.09	0.24	-0.03	0.73
Total cholesterol	-0.003	0.96	0.13	0.08	-0.17	0.045
Iron	-0.17	0.003	-0.11	0.17	-0.24	0.005
TIBC	0.0005	0.99	0.03	0.73	0.001	0.99
Transferrin saturation	-0.10	0.10	-0.03	0.68	-0.18	0.047
Ferritin	0.08	0.19	0.10	0.21	0.05	0.54
Erythropoietin resistance index	0.10	0.09	0.08	0.30	0.13	0.16
Echocardiographic values^a^						
LV mass index	0.12	0.08	0.06	0.53	0.20	0.04
LA diameter	0.11	0.16	0.008	0.94	0.21	0.06
LV ejection fraction	-0.11	0.11	-0.01	0.92	-0.26	0.02
E/E’ ratio	0.03	0.75	0.04	0.75	0.02	0.90

RDW, red blood cell distribution width; CRP, C-reactive protein; TIBC, total iron binding capacity; LV, left ventricle; LA, left atrium, E/E’ ratio, early mitral inflow velocity to peak mitral annulus velocity ratio.

^a^Subjects with echocardiographic measurements; Total population, n = 235; RDW-decreased group, n = 126; RDW-increased group, n = 109

### Composite of CV events and death

**Fig 1:**
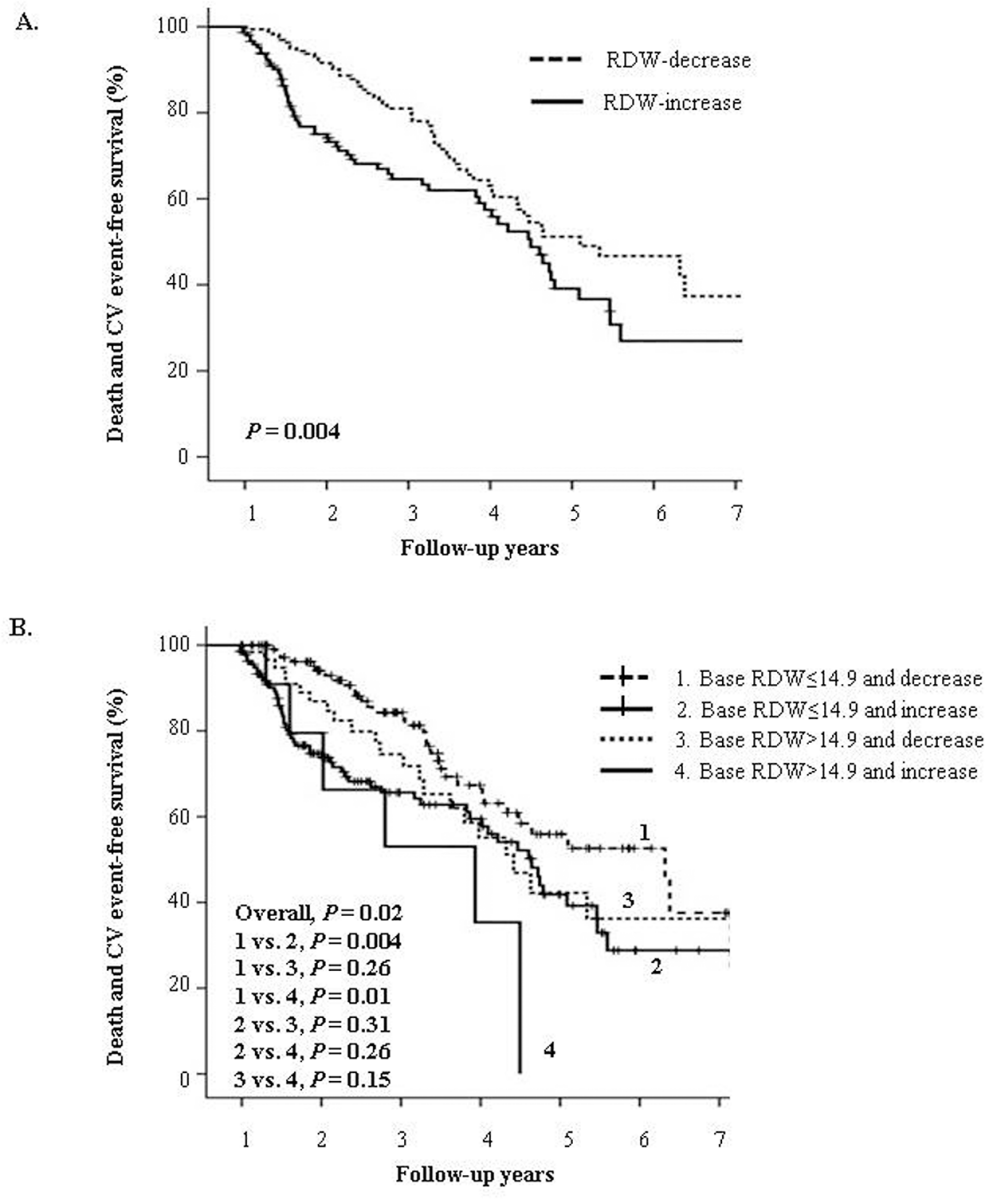
Kaplan-Meier plots for all-cause mortality and nonfatal cardiovascular events. **A.** Comparison of patients according to the change in RDW. The RDW-increased group showed significantly lower event-free survival rates compared to the RDW-decreased group (*P* = 0.004). **B.** Comparison of patients according to the baseline RDW and change in RDW. Patients with baseline RDW>14.9 and RDW increase showed the lowest event-free survival rates, and patients with baseline RDW≤14.9 and RDW decrease showed the highest (*P* = 0.02).

During the follow-up, 75 deaths (24.0%) and 60 non-fatal CV events (18.4%) occurred. The RDW-increased group showed significantly lower event-free survival rates for all-cause death and non-fatal CV events than did the RDW-decreased group (*P* = 0.004; Fig 1A).

Event-free survival rates for composite of end-points were compared after combining two factors: the baseline RDW value and change in RDW. As shown in Fig 1B, patients were subdivided into four groups: patients with baseline RDW ≤14.9% and RDW decrease (n = 115), those with baseline RDW ≤14.9% and RDW increase (n = 138), those with baseline RDW >14.9% and RDW decrease (n = 62), and those with baseline RDW >14.9% and RDW increase (n = 11). Patients with a baseline RDW >14.9% and RDW increase had the lowest event-free survival rates (*P* = 0.02). In the same baseline RDW ≤14.9% group, event-free survival rate was lower in the patients with an RDW increase than in those with an RDW decrease (*P* = 0.004). The event-free survival rates did not differ between patients with a baseline RDW ≤14.9% and RDW increase and those with a baseline RDW >14.9% and RDW decrease (*P* = 0.31).

### Risk analysis of the composite of end-points

The predictors of death and non-fatal CV events were evaluated (Table 4). Univariate Cox regression analysis revealed increased risk for composite end-points in RDW-increased group (Model 1, HR = 1.76, *P* = 0.004). The RDW-increased group remained as a significant predictor after adjustments for demographic and time-averaged laboratory parameters and baseline and follow-up RDW at 1 year (Model 2, HR = 1.75, *P* = 0.007). This significant association remained robust after adjustments for haemoglobin variability measurements (Model 3, HR = 1.75, *P* = 0.007), and after adjustment for vitamin B12 and folate levels (Model 4, HR = 1.72, *P* = 0.04).

**Table 4:** Cox proportional hazard analysis for nonfatal CV events and deaths.

	RDW-increased group (vs. RDW-decreased group)		
	HR	95% confidence interval	*P*
Model 1	1.76	1.20, 2.59	0.004
Model 2	1.75	1.17, 2.61	0.007
Model 3	1.75	1.17, 2.61	0.007
Model 4	1.72	1.03, 2.90	0.04

RDW, red blood cell distribution width.

Model 1: unadjusted relative risk. N = 326.

Model 2: adjusted for for age, sex, diabetes, previous CV disease, baseline RDW and follow-up RDW at 1 year, and time-averaged haemoglobin, log CRP, intact PTH, iron, TIBC and ferritin levels. N = 280.

Model 3: adjusted for Model 2 plus SD, CV, and residual SD of haemoglobin levels, haemoglobin-slope, and RDW-slope. N = 280.

Model 4: adjusted for Model 3 plus vitamin B12 and folate levels. N = 155.

## Discussion

This study showed that a progressive rise in RDW independently predicted all-cause mortality and CV events in ESRD patients. These associations remained significant after adjustments for markers of anaemia, nutrition, haeomoglobin variability, baseline and follow-up RDW, and other CV risk factors, and after subdividing the patients according to the baseline RDW value and change in RDW. These findings suggest that monitoring changes in RDW may be helpful for identifying patients with a high risk of death or CV events after dialysis, even in those with a relatively normal RDW value at the start of dialysis.

Previous studies have shown that RDW is a predictor of CV morbidity and mortality in various populations [2–8]. It was also reported that RDW predicts mortality in acute kidney injury patients treated with continuous renal-replacement therapy [15]. The relevance of RDW to CV morbidity and mortality in ESRD patients has been reported rarely. One study reported that RDW predicted mortality in HD patients, but the number of patients was small [16]. Peng, *et al*. recently reported that a higher baseline RDW value was associated with CV mortality in incident PD patients [17]. Those results differ from our findings, because we found that the baseline RDW was not associated with adverse outcomes. The difference may be related to the number and characteristics of the study population. The study by Peng, *et al*. included a large number of PD patients [17], whereas we included both HD and PD patients, and our sample size was smaller.

Most of the earlier studies derived the RDW value from a single time point rather than investigating the effects of changes in RDW. Acute changes in RDW may result from blood loss or haemolysis, and the RDW value may fluctuate during the early post-dialysis period because of various conditions that are present at the start of maintenance dialysis. Therefore, a single measurement of RDW may be less accurate for making a long-term prognosis. Our study showed that a progressive rise in RDW was independently associated with mortality and CV events in ESRD patients. Similarly, Cauthen, *et al*. showed that a rising RDW value was associated with disease progression in a large number of heart failure patients and that the patients in the lowest RDW tertile had a poor prognosis when their RDW rose [18]. These findings imply that a rising RDW indicates a poor prognosis in patients with a high CV risk such as those with ESRD or heart failure.

Several mechanisms can be postulated to explain the association between rising RDW and adverse outcomes. First, elevated RDW may reflect impaired iron metabolism. In this study, time-averaged RDW values correlated negatively with haemoglobin and iron levels in the total population. The time-averaged RDW values correlated negatively with the levels of haemoglobin and iron, and transferrin saturation in the RDW-increased group, but RDW did not correlate significantly with serum ferritin level. Our results are consistent with previous reports in heart failure patients [2, 19]. These findings suggest that rising RDW is associated with decreased functional iron availability rather than with iron stores. Impaired functional iron availability may affect adverse outcomes in ESRD patients.

The second mechanism may involve inflammatory stress. In this study, time-averaged RDW correlated positively with the log of the CRP concentration in the total population, and the correlation was stronger in the RDW-increased group. Similarly, RDW was positively associated with CRP level in a study based on a nationwide database of people with diabetes [6]. A study of heart failure patients found a positive relationship between elevated RDW and serum levels of interleukin 6, an inflammatory cytokine [2]. Inflammatory cytokines may directly inhibit erythropoietin-induced erythrocyte maturation [20, 21], which leads to an increase in RDW. It is also known that inflammatory cytokines, such as interleukin 1 or interleukin 6, upregulate hepcidin [22], which regulates iron homeostasis by inhibiting iron absorption from the intestine and iron release from reticuloendothelial stores [23].

The third mechanism may involve chronic hypoxia or endothelial dysfunction. In this study, the RDW-increased group showed significant correlations between the time-averaged RDW and LV mass index or LV ejection fraction. Increased RDW is an index of greater variation in erythrocyte volume and is associated with decreased erythrocyte deformability, which can impair blood flow through the microcirculation [24]. The resultant hypoxia may partially contribute to an increase in cardiac load and increased risk for CV events. Endothelial dysfunction may contribute to LV hypertrophy in HD patients [25], and RDW is associated with endothelial dysfunction in non-dialysis chronic kidney disease patients [26].

In this study, patients in the RDW-increased group were older and the percentage of patients who received iron replacement was lower than did those in the RDW-decreased group. The positive relationship between RDW and age is consistent with previous reports [2, 4, 6, 17–19]. This relationship may reflect the fact that elderly people are more likely than younger people to have a nutritional deficiency, comorbidities, and an inflammatory state. Less iron replacement may cause iron deficiency and lead to impaired erythrocyte maturation and a subsequent increase in RDW. However, the ferritin level did not differ between the two groups, suggesting that their iron stores did not differ. It is unclear whether iron replacement affected the change in RDW or whether the RDW-increased group had less iron replacement because of intolerance to oral iron or other medical conditions.

Haemoglobin variability is known to be associated with mortality in ESRD patients [12]. Unexpectedly the RDW-increased group showed lower SD, CV, and residual SD of haemoglobin compared to the RDW-decreased group, which suggests that the RDW-increased group had less fluctuation in haemoglobin levels. The reason is unclear, since multiple factors contribute to haemoglobin variability, including drugs, patient demographics, iron deficiency, infection, and inflammation [11]. We speculate that it may be a reflection of the lower increment in haemoglobin levels in the RDW-increased group. The RDW-increased group was more likely to have lower haemoglobin-slope and higher erythropoietin resistance index compared to the RDW-decreased group. As both groups showed positive haemoglobin-slopes, less increment in haemoglobin level might be represented as better control of haemoglobin variability.

Previous studies have reported that RDW correlated negatively with markers of nutrition, such as albumin, prealbumin, transferrin, and total cholesterol levels, in heart failure patients [27] and in PD patients [17]. This study showed a consistent finding as the total cholesterol level significantly correlated with RDW in the RDW-increased group. However, the serum albumin level did not differ between the two groups, and RDW did not correlate significantly with albumin level. This difference between studies may relate to the fact that long-term nutritional changes were not assessed in our study. Because the serum albumin level was time-averaged values over the year of dialysis, which is a relatively short duration, the values may not be representative of the long-term nutritional state of our study population.

There are several limitations to this study. First, it was a retrospective analysis from a single-centre pool of patients. Second, the potential mechanism for the association between the rise in RDW and poor prognosis was not elucidated. Consecutive measurements of biomarkers of inflammation or nutrition, or echocardiographic variables are warranted to confirm this association. However, the novelty of our study is that we used consecutive RDW measurements to assess the change in RDW, and we found that even in patients with lower baseline RDW values, rising RDW indicated poor prognosis. Therefore, we speculate that a progressive rise in RDW may be a potential predictor for mortality and CV events in ESRD patients.

In conclusion, a progressive rise in RDW predicted all-cause mortality and CV events in ESRD patients, independent of indices of anaemia, nutrition, haeomoglobin variability and traditional CV risks, as well as the baseline and follow-up RDW. RDW is a widely available and inexpensive test performed as part the complete blood cell count. The prognostic strength of a rising RDW may be greater than other expensive and clinically inaccessible markers. Therefore, monitoring the changes in RDW could be an additive predictor for adverse CV outcomes in ESRD patients.
